# UGT72, a Major Glycosyltransferase Family for Flavonoid and Monolignol Homeostasis in Plants

**DOI:** 10.3390/biology11030441

**Published:** 2022-03-14

**Authors:** Nathanaël Speeckaert, Mondher El Jaziri, Marie Baucher, Marc Behr

**Affiliations:** Laboratoire de Biotechnologie Végétale, Université libre de Bruxelles (ULB), Rue des Professeurs Jeener et Brachet 12, 6041 Gosselies, Belgium; nathanael.speeckaert@ulb.be (N.S.); mondher.el.jaziri@ulb.be (M.E.J.); marc.behr@ulb.be (M.B.)

**Keywords:** UGT72 family, glycosylation, flavonoid, monolignol, lignin, plant development

## Abstract

**Simple Summary:**

Phenylpropanoids are specialized metabolites playing crucial roles in plant developmental processes and in plant defense towards pathogens. The attachment of sugar moieties to these small hydrophobic molecules renders them more hydrophilic and increases their solubility. The UDP-glycosyltransferase 72 family (UGT72) of plants has been shown to glycosylate mainly two classes of phenylpropanoids, (i) the monolignols that are the building blocks of lignin, the second most abundant polymer after cellulose, and (ii) the flavonoids, which play determinant roles in plant interactions with other organisms and in response to stress. The purpose of this review is to bring an overview of the current knowledge of the UGT72 family and to highlight its role in the homeostasis of these molecules. Potential applications in pharmacology and in wood, paper pulp, and bioethanol production are given within the perspectives.

**Abstract:**

Plants have developed the capacity to produce a diversified range of specialized metabolites. The glycosylation of those metabolites potentially decreases their toxicity while increasing their stability and their solubility, modifying their transport and their storage. The UGT, forming the largest glycosyltransferase superfamily in plants, combine enzymes that glycosylate mainly hormones and phenylpropanoids by using UDP-sugar as a sugar donor. Particularly, members of the UGT72 family have been shown to glycosylate the monolignols and the flavonoids, thereby being involved in their homeostasis. First, we explore primitive UGTs in algae and liverworts that are related to the angiosperm UGT72 family and their role in flavonoid homeostasis. Second, we describe the role of several UGT72s glycosylating monolignols, some of which have been associated with lignification. In addition, the role of other UGT72 members that glycosylate flavonoids and are involved in the development and/or stress response is depicted. Finally, the importance to explore the subcellular localization of UGTs to study their roles in planta is discussed.

## 1. Introduction

In order to adapt to terrestrial environments and biotic interactions, plants have developed the capacity to produce a wide range of specialized compounds. These compounds can undergo various chemical modifications, such as hydroxylation, methylation, or glycosylation. This capacity has been allowed by the diversification of enzymes able to add new functional groups to molecules. Among these modifications, the glycosylation reaction can occur on –OH, –COOH, –NH2, –SH, and C–C groups in diverse molecules such as proteins, carbohydrates, primary and specialized metabolites, as well as xenobiotics [1]. Glycosylation reaction can be carried out with one or several sugars (oligosaccharides) moieties from activated donors, which generate a great diversity of glycosylated compounds. This conjugation reduces the toxicity of the substrate and increases its stability and its solubility owing to the high polarity of the sugar moiety [1]. Glycosylated compounds are stored mainly in the vacuole and their activity may be restored by a single deglycosylation step when required [2,3]. In addition, glycosylation of molecules can modify their interaction with signal receptors, transport proteins, or degradation systems [4,5].

The ubiquitous glycosyltransferases (GTs) are classified in 114 superfamilies on the basis of the similarity of their amino acid sequences [6,7]. The proportion of *GT* genes in each GT superfamily differs among organisms in function of the peculiarities of their metabolism [8]. GT1, comprising UGTs, is the largest GT superfamily in plants and clusters enzymes glycosylating low molecular weight molecules and making a β-bond using UDP-sugar as sugar donor. *UGTs* represent about 25% and 35% of *GT* genes in *Arabidopsis thaliana* and in *Oryza sativa*, respectively [8]. The diversity of UGTs allows plants to produce and regulate a myriad of specialized metabolites [9,10].

**Figure 1:**
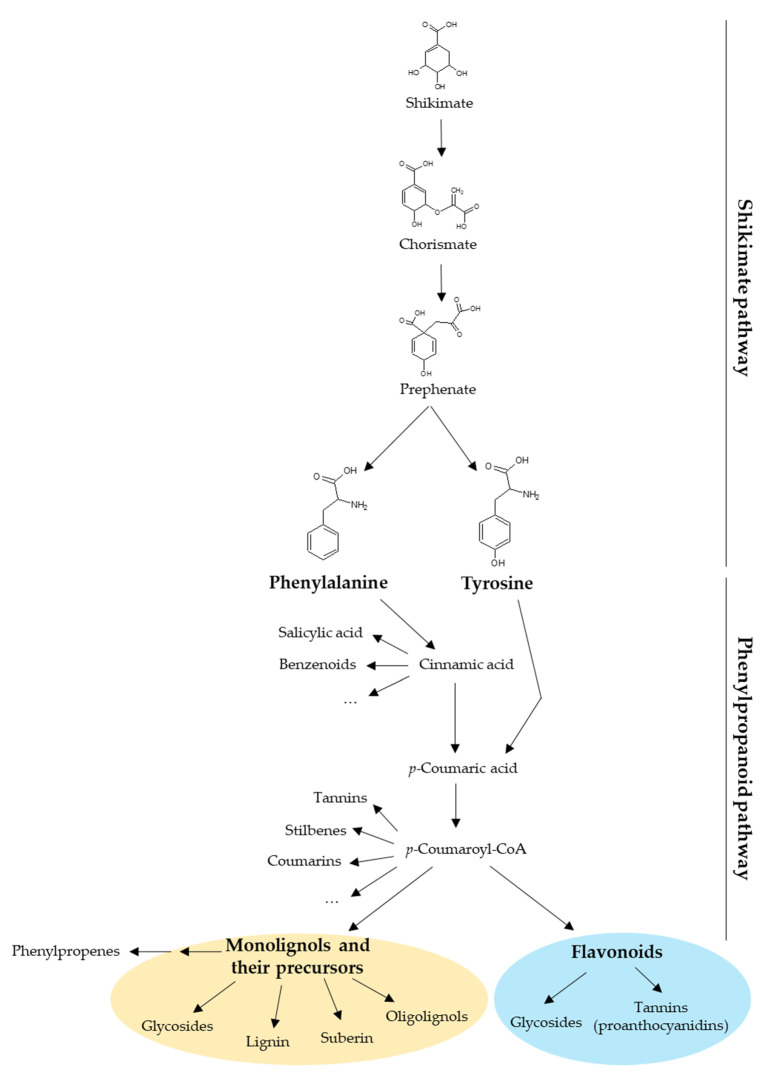
Diversity of compounds produced by the shikimate and the phenylpropanoid pathways in plants. The shikimate pathway yields phenylalanine and tyrosine, which are the precursors of phenylpropanoids. This review focuses on two phenylpropanoid families: the monolignols (yellow circle) and the flavonoids (blue circle). CoA, coenzyme A.

Plant UGTs can glycosylate phytohormones such as abscisic acid, auxins, brassinosteroids, and salicylic acid, as well as terpenoids. However, most of their substrates derive from the phenylpropanoid pathway whose precursors are the aromatic amino acids phenylalanine and tyrosine (Figure 1) [11]. Besides being building blocks for proteins, these two amino acids are also precursors of a large number of plant products with specific functions in plant growth, development, and stress response [12,13]. Phenylalanine and tyrosine are generated by the shikimate pathway in bacteria, fungi, and plants [12,14]. In plants, the shikimate pathway takes place mainly in the chloroplastic compartment and the produced amino acids are exported to the cytosol [12,13]. The first step of the phenylpropanoid pathway is the deamination of phenylalanine by the phenylalanine ammonia-lyase (PAL) producing cinnamic acid. The second step is the hydroxylation of cinnamic acid at the C_4_ position (aromatic nucleus) by cinnamate 4-hydroxylase (C4H) leading to *p*-coumaric acid [15]. Alternatively, tyrosine can be deaminated by bifunctional phenylalanine/tyrosine ammonia-lyase (PTAL) in some plant species, which produces both cinnamic acid from phenylalanine and *p*-coumaric acid from tyrosine [16]. During the third step, a coenzyme A (CoA) is added to *p*-coumaric acid by a 4-coumarate: CoA ligase (4CL) to form *p*-coumaroyl-CoA [15,17,18], which is the last common precursor of monolignols, stilbenes, coumarins, and flavonoids (Figure 1) [19]. In addition, phenylpropenes are synthetized by acetylation and subsequent reduction of monolignols [20]. All these compounds may be glycosylated by UGTs [11]. Monolignol biosynthesis and polymerization have been extensively studied because they are the main components of lignin [2]. In that context, monolignol glycosylation by UGTs may regulate their flux to the cell wall and therefore lignification.

**Figure 2:**
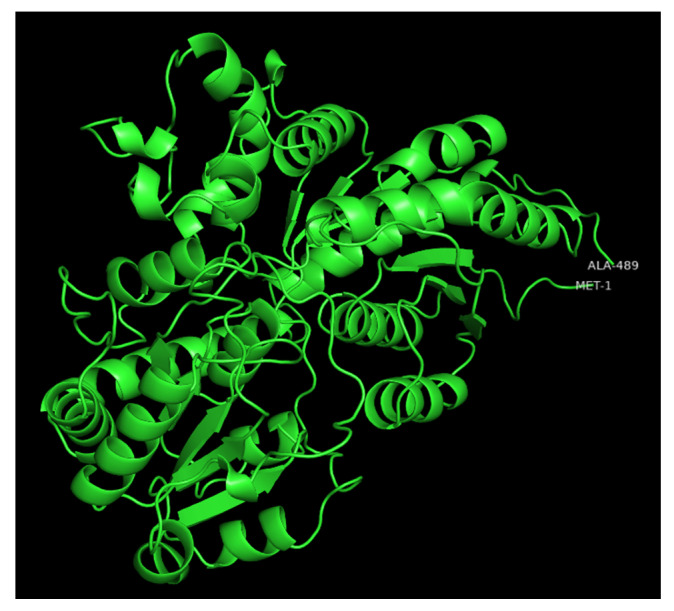
Modeled 3D structure of poplar UGT72A2 obtained with the intensive mode of the Phyre2 web portal [28] and visualized with PyMOL2. The first and last residues are indicated on the polypeptide chain.

UGTs are classified into families in which members share more than 45% of amino acid sequence identity [21]. However, the prediction of substrate specificity based on primary sequence is complex as divergent families can recognize common substrates and closely related UGTs can have different substrate affinities [5,22]. X-ray crystal three-dimensional (3D) structures of a number of plant UGTs have been reported [23,24] and show that their secondary and tertiary structures are conserved [5,25,26]. As an example of the various regions of UGTs, a modeled 3D structure of the poplar UGT72A2 [27] is depicted in Figure 2.

Plant UGTs display a typical GT-B fold which consists of two Rossman fold domains connected by a linker and forming a cleft [7]. The C-terminal domain contains a conserved motif involved in most of the interactions with the UDP-sugar donor. In plants, this consensus sequence is named plant secondary product glycosyltransferase (PSPG) domain and is composed of 44 amino acids [29] whose 10 have a well-known role in the fixation of the UDP-sugar, mainly UDP-glucose [5,30,31,32,33]. In contrast, the acceptor molecule binding site, mainly composed of N-terminal residues, is more variable than the C-terminal part [21]. Different amino acids maintain a hydrophobic environment in the pocket and form a deep narrow cleft. For instance, in the UGT72B1 structure, the substrate is enclosed by six hydrophobic residues (I-86, L-118, F-119, F-148, L-183, and L-197), in addition to E-83 which closes the catalytic site [33]. The correct positioning of the substrate in the pocket is crucial for its glycosylation. The functional group of the sugar acceptor must indeed be close to the first carbon of the sugar, where the link has to be made [5,34]. According to the UGT72B1 model, the H-19 residue is positioned near the sugar acceptor to act as a Brønsted base and deprotonates it before glycosylation, while Q-389 interacts with the glucose moiety of the UDP-sugar [33]. We have recently published a molecular docking summarizing the main interactions between these residues and selected flavonoids using poplar UGT72A2 as a model [27]. According to the opening mechanism of the cleft and the importance of the positioning of the substrates, the physicochemical information about substrates (size, functional group, hydrophobicity, etc.) allows predicting their recognition by UGTs, as well as the regiospecificity of the enzyme [22]. This review focuses on the UGT72 family, whose members are known for their role in the homeostasis of two groups of compounds, the monolignols and the flavonoids (and their derivatives), and thereby on plant development [35,36,37,38,39,40].

## 2. Diversification of UGTs from Algae to Vascular Plants

About 450 million years ago, embryophytes, deriving from green algae emerged from the aquatic habitat and colonized the terrestrial environment thanks to the acquisition of novel biochemical pathways, phytohormones, and signaling pathways [41,42]. Especially, the number of genes coding for enzymes involved in secondary metabolism through the evolution of embryophytes significantly expanded, notably within the families of cytochromes P450, glycosyl hydrolases, and glycosyltransferases [43].

As shown in Table 1, the number of *UGT*s increased during evolution, consisting of five in *Chlamydomonas reinhardtii* to more than 200 in trees such as *Pinus taeda* (gymnosperm) and *Populus trichocarpa* (angiosperm). This divergence suggests an early and continuous expansion of the UGT family in plants which may be related to their constant adaptation to the terrestrial environment and biotic interactions by producing and regulating specialized metabolites [9,10]. Across the embryophytes evolution and especially in gymnosperms and angiosperms, several whole-genome and tandem duplication events happened, diversifying some gene families such as *UGTs* and leading to sub- and neo-functionalization [8,44].

**Figure 3:**
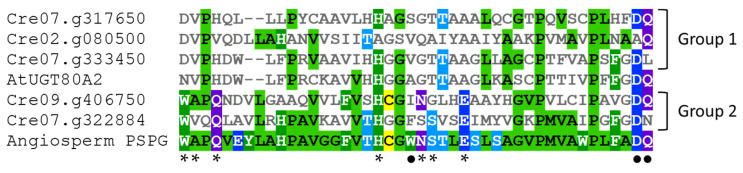
PSPG sequences alignment of the five *C. reinhardtii* UGTs with the PSPG sequence of the Arabidopsis UGT80A2 and the PSPG consensus sequence of angiosperm UGTs (according to [44]). The sequences were aligned using MView [53]. Amino acids are colored depending on their similarity. *: well-conserved amino acids with known function on UDP-sugar binding and enzyme conformation [5,31,32,33]. •: well-conserved amino acids with known function on UDP-glucose recognition [30,31].

An alignment of the PSPG domain of the five UGTs from *C. reinhardtii* with the PSPG consensus sequence for angiosperm UGTs (from 246 protein sequences according to [47]) allows distinguishing two UGT groups in green algae (Figure 3). The PSPG sequences of the group 1 (Cre02.g080500, Cre07.g317650 and Cre07.g333450) show few similarities compared to the angiosperm PSPG consensus and, among the 10 residues with a well-known role in UDP-sugar fixation, only H-19, D-43 (for Cre07.g317650 and Cre07.g333450) and Q-44 (for Cre02.g080500 and Cre07.g317650) are conserved. The H-19 is known to bind to UDP phosphates [32], whereas the last D and Q residues make a hydrogen bond with glucose and are involved in UDP-glucose recognition [30,31,33]. Cre07.g333450, named UGT711A1, is close to the angiosperm UGT80 family (Figure 3) and to fungi UGT families (UGT51-UGT57 and UGT60) presenting a sterol β-glucosyltransferase or a putative sterol β-glucosyltransferase activity [48,49], suggesting that this *C. reinhardtii* UGT recognizes sterols. Glycosylation of sterols is involved in the regulation of the structure and the permeability of the plasma membrane [50,51].

In contrast, the PSPG sequences of the UGTs of *C. reinhardtii* from group 2 (Cre07.g322884 and Cre09.g406750) present most of the amino acids required for UDP-sugar fixation. However, together with the members of group 1, these two UGTs lack W-22 (involved in UDP-glucose recognition), suggesting different sugar donor specificities for these enzymes [31,52]. Other variations such as the absence of S-24 in Cre09.g406750 and of A-2, N-23, and Q-44 in Cre07.g322884 reveal some structural and sugar specificity differences in these green algae UGTs as compared to the majority of angiosperm UGTs [31,32]. For instance, the presence of an N as the last residue of the PSPG domain may allow the binding of UDP-rhamnose as demonstrated for the Arabidopsis UGT78D1 [31].

**Figure 4:**
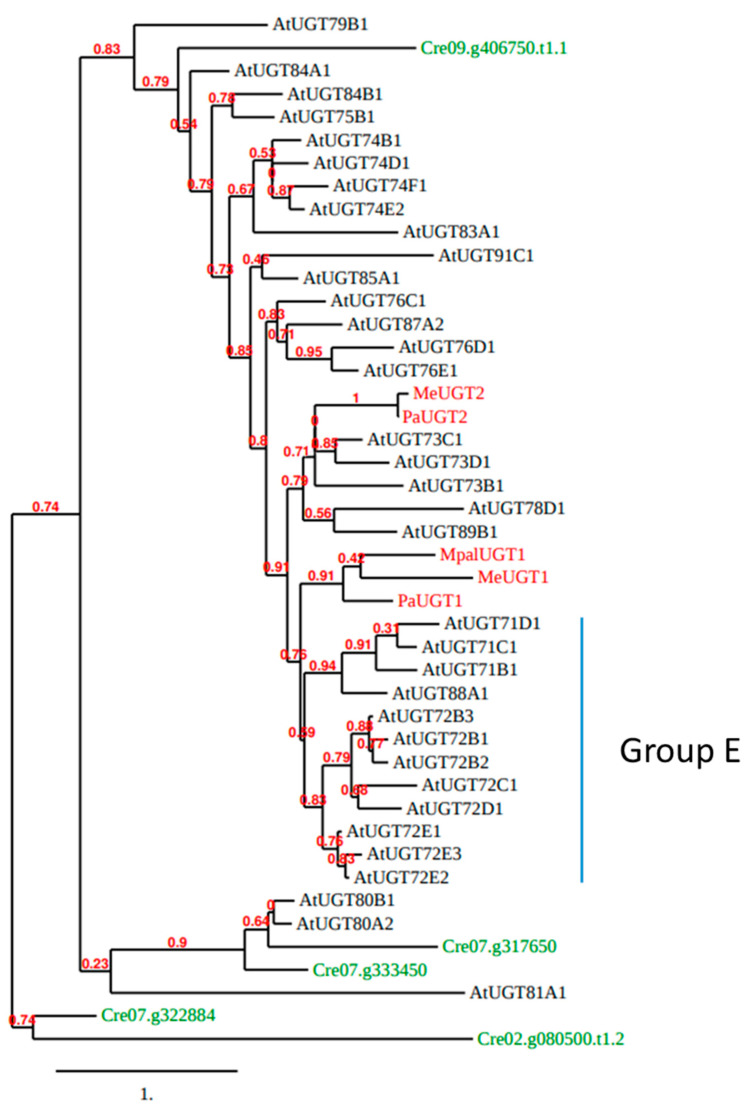
Phylogenetic tree of whole polypeptide sequences of Arabidopsis UGTs (black), five functionally described UGTs (MeUGT1, MeUGT2, MpalUGT1, PaUGT1, and PaUGT2) from 3 species of liverworts (*M. emarginata*, *M. paleacea*, and *P. appendiculatum*; red) and the five *C. reinhardtii* UGTs (green). This tree was generated by the approximate likelihood-ratio test method [56,57]. Scale bar: expected number of amino acid substitutions per site.

To investigate further the evolution of UGT72s, a phylogenetic analysis of Arabidopsis UGTs was performed (Figure 4). We included one representative of each UGT subfamily, but all UGT72s, as well as the five UGTs of *C. reinhardtii* and five functionally described UGTs from liverworts (MeUGT1 and MeUGT2 from *Marchantia emarginata*, MpalUGT1 from *M. paleacea*, as well as PaUGT1 and PaUGT2 from *Plagiochasma appendiculatum*) [54,55]. According to Figure 4, Cre07.g333450 and Cre07.g317650 (Group 1 from Figure 3) are clustered with Arabidopsis UGT80s and Cre09.g406750 (Group 2 from Figure 3) is the only green algae UGT that clusters with all other Arabidopsis UGTs. The liverwort MeUGT1, MpalUGT1, and PaUGT1 classify with the group E of Arabidopsis UGTs (gathering UGT71, UGT72, and UGT88 families according to [21]), while MeUGT2 and PaUGT2 are closer to UGT73s.

The five UGTs from liverwort glycosylate flavonoids. MeUGT1 and MpalUGT1 glycosylate four flavonols (quercetin, kaempferol, isorhamnetin, and myricetin) to form 3-*O*-glucosides [55]. PaUGT1 glycosylates quercetin, kaempferol, and isorhamnetin, the flavones apigenin, luteolin, chrysoeriol, and diosmetin, the flavanones naringenin and eriodictyol, and the dihydrochalcone phloretin to form mainly 7-*O*-glucosides, but also 3-*O*-glucosides [54]. MeUGT2 and PaUGT2 also glycosylate flavonoids [54,55]. Among flavonoids, UGT72s (see the next chapter) glycosylate quercetin, kaempferol, myricetin, apigenin, luteolin, and naringenin indicate that flavonoid glycosylation may be conserved as an ancestral function in plant UGTs.

## 3. Functional Characteristics of the UGT72 Family

### 3.1. Substrate Identification of UGT72s

Table 2 summarizes the different known in vitro substrates reported for 30 recombinant UGT72 proteins distributed into 12 different angiosperm species. UGT72 members recognize flavonoids, monolignols, and their precursors/derivatives as substrates. In addition, several of them *O*/*N*-glycosylate xenobiotics such as chlorophenols and chloroanilines. UGT phylogeny, based on sequence identities and substrate specificities of the enzymes, is not correlated as diverging UGTs can recognize identical substrates. However, the analysis of the 3D structure of the enzyme may predict the substrate specificity [5].

Concerning monolignols and/or their precursors or derivatives, the *A. thaliana* UGT72E1 and UGT72E2 use coniferaldehyde and sinapaldehyde, and UGT72E2 and UGT72E3 glycosylate ferulic acid, sinapic acid, caffeic acid, coniferyl alcohol, and sinapyl alcohol [58]. In addition, UGT72E2 can also glycosylate *p-, m-*, and *o-*coumaric acid, as well as vanillin [58,59]. UGT72B1 glycosylates coniferaldehyde, *p*-coumaraldehyde, coniferyl alcohol, *p*-coumaryl alcohol, and vanillin, whereas UGT72B3 uses coniferaldehyde and sinapaldehyde [39,59]. The *Camellia sinensis* UGT72AM1 glycosylates coniferaldehyde (Table 2) [60]. In *Populus tremula x P. alba*, UGT72AZ2 glycosylates ferulic acid and sinapic acid, UGT72B37 uses *p*-coumaraldehyde, coniferaldehyde, sinapaldehyde, coniferyl alcohol, and sinapyl alcohol, while UGT72B39 glycosylates coniferyl alcohol [61]. In *Solanum lycopersicum*, SlUGT5 glycosylates cinnamyl alcohol [62]. Finally, the *Vanilla planifolia* UGT72U1 and the *Vitis vinifera* UGT72B27 glycosylate vanillin [63,64]. Although not always tested (Table 2), UGT72s glycosylate monolignols and their precursors/derivatives at the C_4_ position on the contrary to UGT84s which glycosylate hydroxycinnamic acids on the alcohol group to form esters as demonstrated in [35].

Concerning flavonoids, the most frequently reported in vitro substrates for UGT72 members are the flavonols quercetin, kaempferol, and myricetin (Table 2). However, the glycosylation pattern can be different among UGT72s, as LjUGT72AH1, LjUGT72Z2, LjUGT72V3, GmUGT72X4, and GmUGT72Z3 form 3-*O*-glycosides [65,66], while MtUGT72L1 forms 3′-*O*-glycoside [67]. In addition, LjUGT72AD1 forms both 3-*O*-glycosides and 7-*O*-glycosides, while CsUGT72AM1 and HpUGT72B11 perform multi-site glycosylation on 3, 7, and 4′ position [60,66,68].

**Table 2:** Known in vitro substrates of 30 recombinant UGT72 proteins distributed into 12 angiosperm species. The regiospecificity of the glycosylation is noted between brackets when determined. All UGT72s use UDP-glucose as sugar donor. In addition, UGT72B1 uses UDP-5-thioglucose [22], UGT72B3, GmUGT72X4, and GmUGT72Z3 use UDP-galactose [22,65] and UGT72B3 uses UDP-xylose [22]. CTP, chlorothiophenol; DCA, dichloroaniline; DCP: dichlorophenol; DHCA: dihydroconiferyl alcohol; HPPA: hydroxyphenylpyruvic acid; TCA: trichloroaniline; TCP: trichlorophenol; TFMP, trifluoromethylphenol. -: no substrate known.

UGT72	Accession	Species	Monolignol Pathway	Flavonoids	Other Phenolics	Other Compounds	References
UGT72B1	At4G01070	*A. thaliana*	Coniferaldehyde *p*-coumaraldehyde Coniferyl alcohol DHCA *p*-coumaryl alcohol Vanillin	-	Umbelliferone 4-methyl-umbelliferone Scopoletin (7-*O*) Esculetin (6-*O*) Hydroxybenzoic acids (3-*O,* 4-*O,* 5-*O*) 4-HPPA 4-nitrophenol Phenol 2,4-DCP 3,4-DCP 2,4,5-TCP 2,3,4-TCP 2,3,6-TCP 2-chloro-4-TFMP 1-naphthol Triclosan Alternariol	3,4-DCA 2,4-DCA 2,4,5-TCA 3,4,5-TCA Picloram	[22,33,39,59,69,70,71,72,73]
UGT72B3	At1G01420	*A. thaliana*	Coniferaldehyde Sinapaldehyde	Quercetin Fisetin Kaempferol	4-methyl-umbelliferone Scopoletin (7-*O*) Esculetin (7-*O*) Umbelliferone 4-acetic acid 2,4,5-TCP 2-chloro-4-TFMP	-	[22,39,70,72]
UGT72C1	At4G36770	*A. thaliana*	-	-	Scopoletin (7-*O*) Esculetin (6-*O*) 2,4,5-TCP	-	[22,70]
UGT72D1	At2G18570	*A. thaliana*	-	Luteolin Quercetin Fisetin Kaempferol Taxifolin Catechin Genistein	4-methyl-umbelliferone Scopoletin (7-*O*) Esculetin (6-*O*) Dihydroxylbenzoic acids 2,4,5-TCP	-	[22,70,74]
UGT72E1	At3G50740	*A. thaliana*	Coniferaldehyde (4-*O*) Sinapaldehyde (4-*O*)	Quercetin Fisetin Kaempferol	-	-	[22,36,58]
UGT72E2	At5G66690	*A. thaliana*	Ferulic acid (4-*O*) Sinapyl alcohol (4-*O*) Sinapic acid (4-*O*) Caffeic acid (4-*O*) *p*-coumaric acid (4-*O*) *o*-coumaric acid *m*-coumaric acid Coniferaldehyde (4-*O*) Sinapaldehyde (4-*O*) Coniferyl alcohol (4-*O*) Vanillin	-	Scopoletin (7-*O*) Esculetin (6-*O*) 2,4,5-TCP	3,4-DCA	[22,35,36,58,59,70,75]
UGT72E3	At5G26310	*A. thaliana*	Sinapic acid (4-*O*) Caffeic acid (4-*O*) Ferulic acid (4-*O*) Coniferyl alcohol Sinapyl alcohol	-	Scopoletin (7-*O*) Esculetin (6-*O*)	-	[22,35,36,58,70]
UGT72AM1	KY399734	*C. sinensis*	Coniferaldehyde (4-*O*)	Kaempferol (3-*O*) Quercetin (3-*O*) Myricetin (3-*O*) Naringenin (7-*O*, 4′-*O*) Eriodictyol Dihydromyricetin Cyanidin (3-*O*)	-	-	[60,76]
UGT72X4	GLYMA8G338100	*G. max*	-	Quercetin (3-*O*) Kaempferol (3-*O*) Myricetin (3-*O*)	-	-	[65]
UGT73	GLYMA8G338200	*G. max*	-	Quercetin (3-*O*) Kaempferol (3-*O*) Myricetin (3-*O*)	-	-	[65]
UGT72B11	EU561016	*H. pilosella*	-	Baicalein (7-*O*) Quercetin (3-*O*, 4′-*O*) Kaempferol (3-*O*, 7-*O*) Apigenin (7-*O*) Luteolin (7-*O*, 4′-*O*) Naringenin (7-*O*) Eriodictyol Scutellarein Chrysin Myricetin Morin	Umbelliferone Esculetin Catechol Resorcinol Hydroquinone	-	[68]
UGT72AD1	AP009657	*L. japonicus*	-	Kaempferol (3-*O*, 7-*O*) Quercetin (3-*O*) Myricetin (3-*O*)	-	-	[66]
UGT72AF1	KT895083	*L. japonicus*	-	Apigenin Daidzein Genistein	-	-	[66]
UGT72AH1	AOG18241	*L. japonicus*	-	Kaempferol Quercetin Myricetin	-	-	[66]
UGT72V3	KT895088	*L. japonicus*	-	Kaempferol Quercetin Myricetin Luteolin Daidzein Genistein	-	-	[66]
UGT72Z2	KP410264	*L. japonicus*	-	Kaempferol (3-*O*) Quercetin (3-*O*) Myricetin (3-*O*)	-	-	[66]
UGT72L1	ACC38470	*M. truncatula*	-	Epicatechin (3′-*O*) Epigallocatechin	-	-	[67]
UGT72AX1	Nbv6. 1trP17460	*N. benthamiana*	-	Kaempferol	Carvacrol Hydroquinone Scopoletin Carveol Alternariol	3-*cis*-hexenol 1-octen-3-ol Benzyl alcohol Lavandulol 2-phenylethanol Farnesol Perillyl alcohol β-ionol Geraniol	[73,77]
UGT72AY1	Nbv6. 1trP2283	*N. benthamiana*	-	Kaempferol Malvidin	Carvacrol Hydroquinone Scopoletin Carveol Alternariol	Farnesol α-ionol β-ionol 2-phenylethanol Geraniol 3-*cis*-hexenol 1-octen-3-ol Perillyl alcohol Benzyl alcohol Lavandulol Tyrosol Myrtenol 3-oxo-α-ionol Mandelic acid Mandelonitrile Furanmethanethiol Sotolone Maple furanone Furaneol Homofuraneol	[73,77,78]
UGT72B34	Nbv6. 1trP17549	*N. benthamiana*	-	Kaempferol	Carvacrol Hydroquinone Scopoletin Carveol	Geraniol 3-*cis*-hexenol Perillyl alcohol 1-octen-3-ol Benzyl alcohol Lavandulol Tyrosol Myrtenol Farnesol 2-phenylethanol	[77]
UGT72B35	Nbv6. 1trP72850	*N. benthamiana*	-	Kaempferol	Carvacrol Hydroquinone Scopoletin	Benzyl alcohol Tyrosol Furanmethanethiol α-bisabolol 1-octen-3-ol 2-phenylethanol	[77]
PtGT1	HM776516	*P. tomentosa*	-	-	-	-	[38]
UGT72AZ1	Potri- 7G030300	*P. tremula* *x P. alba*	-	-	-	-	[61]
UGT72AZ2	Potri- 7G030400	*P. tremula* *x P. alba*	Ferulic acid Sinapic acid	-	-	-	[61]
UGT72A2	Potri- 7G030500	*P. tremula* *x P. alba*	-	-	-	-	[27,61]
UGT72B37	Potri- 14G096100	*P. tremula* *x P. alba*	*p*-coumaraldehyde Coniferaldehyde Sinapaldehyde Coniferyl alcohol Sinapyl alcohol	-	-	-	[61]
UGT72B39	Potri- 2G168600	*P. tremula* *x P. alba*	Coniferyl alcohol	-	-	-	[61]
SlUGT5	HM209439	*S. lycopersicum*	Cinnamyl alcohol	Kaempferol	Methyl salicylate Guaiacol Eugenol Hydroquinone Salicyl alcohol (7-*O*)	Benzyl alcohol	[62]
UGT72U1	Not available	*V. planifolia*	Vanillin	-	-	-	[63]
UGT72B27	AM483418	*V. vinifera*	Vanillin	-	Trans-resveratrol (3-*O*, 4′-*O*) Thymol Carvacrol Eugenol Guaiacol 4-methylguaiacol Syringol 4-methylsyringol m-cresol o-cresol Alternariol	Menthol Sotolone Furaneol Homofuraneol	[64,73,78,79]

### 3.2. Possible Roles of UGT72s in Monolignol Homeostasis and in the Regulation of Lignification

#### 3.2.1. Monolignol Homeostasis

The role of *UGT72*s in monolignol homeostasis has been investigated in *A. thaliana* and in poplar. In leaves of *A. thaliana*, the overexpression of *UGT72E2* and *UGT72E3* induces the accumulation of both coniferin and syringin as compared to the wild type where they are not or poorly detected, while the overexpression of *UGT72E1* induces a small accumulation of coniferin. Similarly, in light-grown roots, the overexpression of *UGT72E2* and *UGT72E3* induces an increase in both coniferin and syringin content as compared to the wild type, and the overexpression of *UGT72E1* doubles the amount of coniferin [36,37]. The downregulation of *UGT72E2* induces a decrease in monolignol glucosides content in the light-grown roots as compared to the wild type (Table 3) [36]. However, the simultaneous downregulation of the three Arabidopsis *UGT72E* results in a more pronounced reduction of both coniferin and syringin content than the single *UGT72E2* downregulation, suggesting possible redundancy of their function [36]. Furthermore, the overexpression of *UGT72E2* and *UGT72E3* in Arabidopsis induces a decrease in the amount of sinapoyl malate, as well as the accumulation of ferulic acid glucoside in leaves. In addition, the overexpression of *UGT72E3* induces an accumulation of sinapic acid glucoside. These results show modifications in the allocation of phenylpropanoids following alteration of the expression of UGT72s (Table 3). However, no significant difference was evidenced in the total soluble phenolics content in leaves [36,37].

Because of their roles in monolignol homeostasis, UGT72Es may be involved in the resistance to pathogens. In fact, *A. thaliana* plants overexpressing *UGT72E2* infected with the fungus *V. longisporum* showed a 5-fold increase in the coniferin amount, and their susceptibility to the pathogen was reduced (as the transgenic plants were 2-fold less stunted and as 10-fold less fungal DNA content was detected by real-time PCR, when compared to the wild type; Table 3) [3]. In vitro assays showed that treatment with 100 µM coniferyl alcohol reduces fungal growth which was not observed after a treatment with 100 µM coniferin. However, in the coniferin treatment, the melanization (the fungal last developmental stage) is delayed with only minor black spots compared to the control. As proposed by these authors, coniferin may be hydrolyzed by a *β*-glucosidase into coniferyl alcohol during the cell lysis. Afterwards, coniferyl alcohol may inhibit *V. longisporum* growth and be further oxidized into ferulic acid, which is toxic for the pathogen [3].

The overexpression of *UGT72B1* induces an increase in coniferin content. However, the knock-out mutant of *UGT72B1* also accumulates more coniferin than the wild type (Table 3) [39]. This unexpected result was linked to an increase in the expression of genes of the phenylpropanoid pathway in this mutant (i.e., *CCR2*, *COMT1*, *COMT2*, *HCT2*, *CAD1*, *CAD5*, *CAD8*, *4CL*, *C4H*, *CCoAOMT1*, and *PAL1*). Moreover, *UGT72B3* and *UGT72E2* are also upregulated in the *ugt72b1* mutant and could compensate for the UGT72B1 defection [39]. In that case, UGT72B1 may partake in the gene expression regulation of the phenylpropanoid pathway by a mechanism that remains to be determined. In addition, the mutant exhibits an accumulation of anthocyanins in the shoot tips which is explained by the upregulation of two important genes of the flavonoid biosynthesis, *CHS* and *DFR* [39]. Hence, UGT72B1 appears to have a role in both monolignol and flavonoid homeostasis.

In poplar (*P. tremula × P. alba*), the overexpression of *UGT72AZ1* and *UGT72AZ2* triggers the accumulation of coniferin, and the overexpression of *UGT72AZ1* causes also the accumulation of syringin (Table 3). However, the corresponding recombinant proteins do not use monolignols. In contrast, UGT72B37 and UGT72B39 glycosylate monolignols in vitro, but the overexpression of the corresponding genes in poplar does not result in a higher accumulation of monolignol glucosides [61]. These differences in substrate specificity in vitro and in vivo may be due to the substrate availability in planta.

#### 3.2.2. Regulation of Lignification

The involvement of monolignol glucosylation in lignification has been frequently discussed [80,81,82,83]. On the one hand, this process may have a role in monolignol transport in the cell. The mechanisms of subcellular monolignol transport are still under debate, and indirect experiments have evidenced both passive and ATP-dependent transport [80,81,84,85]. Molecular dynamic simulations support that most of the phenylpropanoids involved in lignification can passively cross the membranes, but not their glycosylated derivatives [81]. An ATP-dependent transport of both monolignols and monolignol glucosides has also been evidenced in isolated membrane vesicles from Arabidopsis [80]. As shown by these authors, only aglycon monolignols can cross the plasma membrane, while only glycosylated monolignols can cross the tonoplast, suggesting that monolignol glucosylation may determine the allocation of monolignols in cells [80]. On the other hand, monolignol glycosides may be directly incorporated into lignin as shown by biomimetic in vitro assays (dehydrogenative polymerization catalyzed by horseradish peroxidase in the presence of coniferin and syringin with or without commercial almond *β*-glucosidase) and nuclear magnetic resonance (NMR) spectroscopy analysis of cell wall and lignin fractions of wood from gymnosperms and angiosperms [83]. This incorporation could possibly intervene into the lignin–carbohydrate complex (linkages between lignin and hemicelluloses/cellulose), which adds complexity for the understanding of the role of glycosylation in the lignification process [83].

Candidates for lignin polymerization regulation have been searched among Arabidopsis and poplar UGT72s that glycosylate monolignols and/or their precursors. In Arabidopsis, GUS assays showed that *pUGT72E2*- and *pUGT72E3*-driven expressions are associated with vascular tissues in seedlings (roots, cotyledons, and apical meristem) and in flowers. In addition, *pUGT72E2-*driven expression is associated with vascular tissues in leaves [37]. *pUGT72B1-*driven expression was mainly found in the developing xylem, the pith, and the cortex of young floral stems while it is only expressed in the xylem of old floral stems (Table 3) [39]. These results suggest that *UGT72E2*, *UGT72E3*, and *UGT72B1* may be associated with vascular tissue development, and possibly with lignification. In addition, the transcriptomic analysis of the triple *lac4 lac11 lac17* Arabidopsis mutant characterized by a highly disrupted lignin deposition revealed a relation between the expression of *UGT72Es* and that of *laccases* (*LAC*) and *peroxidases* (*PRX*) associated with lignin polymerization. In the leaves of this mutant, coniferin and syringin contents are 4-fold higher than the wild type, and the expression of both *UGT72E2* and *UGT72E3* is 5.2-fold and 2-fold increased, respectively [86]. Another transcriptomic study showed that Arabidopsis transgenic lines overexpressing *MYB58* or *MYB63* upregulate monolignol biosynthesis genes as well as *UGT72E2*. These transgenic lines accumulate monolignol glucosides and display ectopic lignification in the epidermis, cortex, and pith [87]. Finally, *PRX49* and *PRX72*, two genes encoding peroxidases likely involved in lignification [88,89], are co-expressed with *UGT72E1/UGT72E2* and with the three *UGT72Es*, respectively [90].

Functional characterization of mutants has evidenced phenotypes to clarify this question. Although single *ugt72e1*, *ugt72e2* or *ugt72e3* Arabidopsis mutants do not show any difference in lignin quantity in the floral stem when using the acetyl bromide method, a higher proportion of lignin in the xylem and interfascicular fiber cell walls of the *ugt72e3* mutant was evidenced by Raman microspectroscopy and safranin O ratiometric imaging technique, as compared to the wild type [40]. This difference was only observed in the young part and not in the old part of the floral stem and indicates a role of UGT72E3 in cell wall lignification during vascular cells development. Moreover, a higher capacity of incorporation of the three fluorescently labeled monolignols into lignin was found in the *ugt72e3* young stem as compared to the wild type. This phenotype was related to an increased expression of lignin-specific *PRX71* and *LAC17* [40]. The *ugt72b1* mutant exhibits a higher lignin content in the whole floral stem, as well as an ectopic lignification in the interfascicular fibers and the pith as compared to the wild type and the rescued line (insertion of the *UGT72B1* cDNA under the control of the native gene promoter) [39]. In addition, the mutation leads to a 4-fold increase in the thickness of the pith cell wall. Like monolignol biosynthesis genes, genes potentially involved in monolignol transport across the plasma membrane and in lignin polymerization were upregulated (i.e., *PRX34*, *PRX37*, *PRX71*, *PER64*, *LAC5*, *LAC12*, *LAC15*, *ABCG29*, *ABCG40*, *RBOHA* and *RBOHD*) [39]. The authors suggest that the *UGT72B1* mutation, which depletes the content of monolignol glucosides, may trigger a signal upregulating the genes involved in monolignol biosynthesis, transport, and polymerization, generating an overproduction of monolignols, hence an increased and ectopic lignification. Consequently, the increase in coniferin in the mutant may be a secondary effect of this monolignol overproduction [39]. The alteration of the monolignol biosynthesis and of lignification may interfere with other developmental processes, explaining the repression of the shoot growth of the *ugt72b1* mutant [39].

In poplar, the *UGT72s* encoding proteins glycosylating monolignols and/or their precursors were expressed in vascular tissues of stem or roots (Table 3). In 4-month-old poplar, *UGT72AZ1* is mainly expressed in phloem of stem and leaf, *UGT72B37* and *UGT72B39* are expressed in the primary xylem and in the secondary xylem of the stem, respectively, and *UGT72AZ2* is expressed in the cortical region, in the phloem, and in the differentiating xylem of the root [61]. However, although the overexpression of *UGT72AZ1* and *UGT72AZ2* in *P. tremula × P. alba* increases the monolignol glucosides content in the leaf, there was no modification of total lignin content in the stem nor in the root [61]. It is possible that the effect of the increase in monolignol glucosides on lignification is compensated by other processes, such as hydrolysis catalyzed by *β*-glucosidases or incorporation of the glucosides in the growing lignin polymer, or that *UGT72AZ1* and *UGT72AZ2* are involved in other processes such as for instance the regulation of oligolignol biosynthesis or the defense towards pathogens. The *P. tomentosa PtGT1* (orthologous to *UGT72AZ2*), when ectopically expressed in tobacco, induces a 60% increase in Klason lignin content in the stem of a 2-month-old plant (Table 3). However, as no enzymatic activity against monolignols was detected for the recombinant PtGT1 (Table 2), the effect of the *PtGT1* ectopic expression on lignin content was suggested to be indirect [38].

### 3.3. UGT72s Involved in Flavonoid Homeostasis

Flavonoids have many roles in plants, especially in stress responses and development [91]. As several UGT72s glycosylate flavonoids, they can be critical in the regulation of these processes. Indeed, glycosylation may modify the activity, transport, accumulation, and biosynthesis of flavonoids [92]. For example, Pang et al. (2008) have demonstrated in vitro that the *M. truncatula* UGT72L1 can 3′-*O*-glycosylate epicatechin and epigallocatechin [67]. Epicatechin and epigallocatechin (as well as catechin) are the main components of proanthocyanidins, or condensed tannins which are found in fruits, flowers, bark, and seeds of many plants and whose astringency is known to protect plants against pathogens and herbivores [93]. Moreover, they provide UV protection and antioxidant activity to the plant and are involved in defense against biotic and abiotic stresses [94]. In the *ugt72l1* mutant, the reduction of both epicatechin and epicatechin 3′-*O*-glucoside content triggers a decrease in extractable proanthocyanidins in seeds. In contrast, the overexpression of *UGT72L1* in *M. truncatula* hairy roots increases both the extractable and non-extractable proanthocyanidins content and decreases the anthocyanins content (Table 4) [95]. GUS assays evidenced that *UGT72L1* is expressed in the mid-rib of the rosette leaves, the peduncles of the siliques and the inflorescence, and developing seeds of *A. thaliana*, in a similar way to genes involved in proanthocyanidins biosynthesis (e.g., *ANR, CHS*, and *TTG1*; Table 4) [67]. UGT72L1 could regulate proanthocyanidin biosynthesis by directing the flux of epicatechin into the vacuole [95]. Indeed, the MATE1 transporter, which is involved in the epicatechin transport across the tonoplast like its Arabidopsis homologous TT12, has been demonstrated to be specific to epicatechin 3′-*O*-glucoside [96]. Moreover, as suggested by Pang and colleagues [67], this glycosylation may protect the plant against free epicatechins and help to direct monomers polymerization into the accurate 4–8 linkage.

The majority of UGT72s glycosylating flavonoids in vitro have not been studied for their role in stress tolerance. However, a transcriptomic analysis reveals, for instance, that *UGT72B3* is upregulated in Arabidopsis aerial parts when temperature decreases from 20 °C to 4 °C, while *UGT72E1* is downregulated in the same conditions. *UGT72E1* and *UGT72D1* are upregulated in roots after infection by *Plasmodiophora brassicae. UGT72D1* is downregulated during drought [97].

In *Populus tremula x P. alba*, *UGT72A2* is mainly expressed in young leaf and stem (Table 4). The downregulation of *UGT72A2* triggers leaf yellowing and necrosis under standard growing conditions in comparison to the wild type [27]. This phenotype was associated with oxidative stress in leaves characterized by higher lipid peroxidation and the decrease in compounds involved in oxidative stress response when compared to the wild type. Especially the total flavonoid content, the anthocyanin content and the total phenolic content in leaves are lower in the *UGT72A2*-depleted lines than in the wild type. Consistently, *pUGT72A2::GUS* was significantly transactivated by the poplar transcription factor MYB119 regulating the biosynthesis of flavonoids. Moreover, the leaves of the *UGT72A2-*depleted lines show a decreased soluble peroxidase activity and a decrease in the NADPH to NADP^+^ ratio, both indicating an alteration of the redox scavenging system [27]. Curiously, the downregulation of *UGT72A2* improves the tolerance to methyl viologen in leaves, an herbicide enhancing the production of superoxide ions in the chloroplasts [27]. This increased tolerance may be linked to the observed accumulation of proanthocyanidins which was previously demonstrated to protect poplar leaves against oxidative stress induced by methyl viologen (Table 4) [27,98]. This functional characterization of *UGT72A2* reveals the function that UGT72s may have on flavonoid homeostasis, as well as on ROS scavenging and stress tolerance.

Flavonoids are also involved in developmental processes, especially by interacting with hormone signalings [91,99] and UGT72s may also be involved in those processes. In *L. japonicus*, UGT72AD1 and UGT72Z2 glycosylate in vitro kaempferol, quercetin, and myricetin, both forming 7-*O*-glycosides, while 3-*O*-glycosides were detected for UGT72AD1 only [66]. They are mainly expressed during the later stages of seed development in a similar manner to *FLS*, *MYB11*, and *MYB14*, which are involved in flavonoid biosynthesis [66]. The overexpression of *UGT72AD1* and *UGT72Z2* in *L. japonicus* hairy roots does not significantly modify the total flavonoid content. However, the flavonol content is increased in all of the transgenic lines compared to the wild type. Especially, kaempferol 3-*O*-rhamnoside-7-*O*-rhamnoside, kaempferol 3-*O*-glucoside-7-*O*-rhamnoside, and two additional flavonol hexosides are more accumulated. These results suggest that UGT72AD1 and UGT72Z2 may be involved in the kaempferol glycosides homeostasis and especially in kaempferol rhamnosides homeostasis (Table 4) [99]. A previous study had shown that increased kaempferol 3-*O*-rhamnoside-7-*O*-rhamnoside content inhibits polar auxin transport, affecting the growth and the gravitropism in Arabidopsis [99]. In accordance, transgenic Arabidopsis lines overexpressing *UGT72AD1* and *UGT72Z2* showed a significant inhibition of root growth (Table 4), suggesting a role of these genes in auxin homeostasis and developmental regulation in *L. japonicus* [66].

### 3.4. The Subcellular Localization of UGT72s Provides Information on Their Functions

UGT72s have been localized in different subcellular compartments. For instance, UGT72L1 fused to the green fluorescent protein (GFP) was detected in the cytosol in *M. truncatula* [95]. The first plant UGT localized into the nucleus was UGT72E1-GFP which interacts with the Arabidopsis MAP kinase kinase kinase SIS8, involved in sugar signaling [100]. In poplar, five UGT72s fused to GFP (UGT72AZ1, UGT72AZ2, UGT72B36, UGT72B37, and UGT72B39) are localized both in the nucleus and associated with the endoplasmic reticulum (ER). The localization of UGT72s in the nucleus may reveal a specialization of these UGTs in the phenylpropanoid homeostasis in this specific compartment. There is no report of monolignol glycosides in the nucleus and their potential function. However, some flavonoids are localized in the nucleus in several plant species, suggesting that this localization is also possible for monolignols [101,102,103]. It has been proposed that flavonoids may interfere with some nuclear proteins involved in DNA organization, signaling pathway, and gene expression [103,104,105], and these processes may be regulated by UGT72s.

The *Polygonum tinctorium* indoxyl-β-D-glucoside synthase (IGS), a member of the UGT72B sub-family, was localized in both cytosolic and microsomal fractions, suggesting a reversible binding of the protein to the membranes. The ER localization was confirmed using ultracentrifugation with a sucrose density gradient [106]. The *Pyrus bretschneideri* PbUGT72AJ2 fused to GFP was also located mainly in the cytosol and cytomembrane compartment [107]. The enzymatic steps of the phenylpropanoid pathway are known to occur in the cytosol and several enzymes of the monolignol biosynthesis pathway, such as HCT and the P450 proteins C4H, C3′H, and F5H are, at least partly, associated with the ER [108,109]. Assembly and disassembly of a given metabolon, for instance, a UGT coupled to a P450 protein would provide additional flexibility in specialized metabolite biosynthesis. Finally, UGT72A2-GFP is localized in the chloroplast [61]. The chloroplast localization of UGT72A2 fits with its function in flavonoid and ROS homeostasis within the chloroplast. Chloroplasts are indeed storage and even biosynthesis organelles for some phenylpropanoids such as kaempferol, quercetin, and catechin in different plant species [110,111,112]. These compounds may be involved in ROS scavenging in this compartment [113]. The subcellular localization of the majority of UGT72s has not been investigated yet, which could improve our knowledge of the biological functions in this family.

## 4. Challenges and Perspectives in UGT Research

Our knowledge of the glycosyltransferase activity of UGT72s remains limited because the enzymatic assays are performed in vitro and do not reflect the availability of the appropriate substrates in planta. In addition, since most of the recombinant proteins are produced using *E. coli* as an expression host, the significance of several post-translational modifications occurring in plants is not adequately addressed. For instance, various patterns of protein *N*-glycosylation modified the affinity of human UGT2B7 towards zidovudine and morphine [114]. Such an experimental limitation may be overcome by studying recombinant proteins in yeast, as shown by the expression of *UGT72E2* and *UGT72B1* in *Saccharomyces cerevisiae* or *Schizosaccharomyces pombe* engineered with the vanillin pathway, thereby allowing to produce vanillin β-D-glucoside which is less toxic than vanillin [59]. Otherwise, the investigated UGT may be extracted from a transgenic plant overexpressing its coding gene and to compare its glycosylation activity to proteins extracted from a control plant. This experimental setup was successfully applied to confirm in planta the activity of UGT92G6 detected in vitro with caffeic acid [115]. Chen and colleagues evidenced the activity of UGT78H2 extracted from transiently modified tobacco leaves towards quercetin [116].

The glycosyltransferase activity of UGT72s against monolignols and flavonoids opens avenues across numerous applications. The glycosylation of these compounds is extensively studied in the food and pharmacology sectors, because the biological functionalities of small molecules may be enhanced by increasing their hydrophilicity and stability through glycosylation [117]. For instance, syringin is known for its anti-inflammatory, anti-nociceptive, immune-modulatory, and anti-diabetic effects [118,119,120]. Despite the biosynthesis pathway of syringin being well conserved in the plant kingdom, few plant species naturally accumulate large quantities of syringin. In order to improve the biosynthesis of syringin in plants, a study has investigated the creation of a chimeric protein between UGT72E2 and UGT72E3 where the high glycosyltransferase activity of UGT72E2 is coupled with the higher affinity for sinapyl alcohol of UGT72E3. When this chimeric *UGT72E3/2* gene is overexpressed in Arabidopsis plants, there is 145% more syringin in leaves than in the transgenic Arabidopsis overexpressing *UGT72E3* [121]. Flavonoids are also important in promoting health molecules because of their antioxidant properties and their beneficial effect against, for instance, hypertension, inflammation, bacterial infections, cerebral disorder, atherosclerosis, and cancer [122]. The purple-leaf tea variety ‘Zijuan’, accumulating high levels of anthocyanidins and anthocyanins, has been selected for its antiproliferative effects on colorectal carcinoma cells [123]. Molecular analyses have shown that *UGT72AM1* is 4.2-fold more expressed in this variety than in the wild type (the ‘Longjing43′ tea variety). As the protein coded by this gene can glycosylate in vitro kaempferol, quercetin, myricetin, naringenin, eriodictyol, and cyanidin, its higher expression may be linked to the higher content of glycosylated flavonoids in the ‘Zijuan’ variety [60,76].

The monolignol biosynthesis pathway and the impact of its modification on biomass recalcitrance is extensively studied to improve cellulose extraction for the production of paper pulp and bioethanol. Actually, lignin is the main limiting factor in these sectors because it restricts polysaccharides’ accessibility and inhibits enzymatic activity. Reducing the lignin content in the cell wall and/or altering its composition are considered as two strategies to improve pulping and saccharification [124]. The investigation of the role of UGT72s in monolignol homeostasis has revealed an additional novel regulation process for lignification in Arabidopsis [39,40]. In poplar, the role of UGT72s in lignin regulation is less clear and is worth further investigation according to the importance of trees in wood, paper pulp, and bioethanol production.

## 5. Conclusions

**Figure 5:**
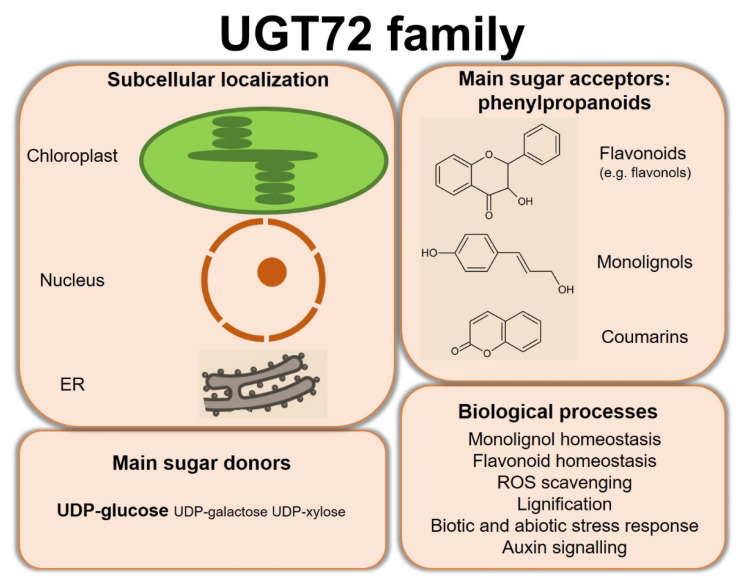
Summary of the subcellular localization and main substrates of UGT72s, as well as the biological processes in which investigated enzymes are involved. ER, endoplasmic reticulum.

Substrates of UGT72 enzymes, using mainly UDP-glucose as sugar donor, cover a wide range of specialized metabolites and xenobiotics. Figure 5 summarizes the main properties of the UGT72 family. In particular, several members of this family have been shown to glycosylate phenylpropanoids including flavonoids, monolignols, and coumarins. Expression and functional studies highlighted a role for these UGT72s in various biological processes such as monolignol homeostasis, flavonoid homeostasis, ROS homeostasis, lignification, response to biotic and abiotic stresses, and auxin signaling. While the subcellular localization of some UGT72s was determined, such as in chloroplast or related to the endoplasmic reticulum, this biological feature is too often not investigated despite its importance regarding the function of the enzyme.

## Figures and Tables

**Table 1:** *UGTs* occurrence in different species from green algae to higher plants.

Taxon	Species	Number of *UGTs*	References
Green Algae–Chlamydomonadaceae	*C. reinhardtii*	5	[45] (Pfam: PF00201)
Marchantiophytes–Marchantiaceae	*Marchantia polymorpha*	41	[41]
Bryophytes–Funariaceae	*Physcomitrella patens*	30	[11]
Lycopodiophytes–Selaginellaceae	*Selaginella moellendorffi*	137	
Gymnosperms–Ginkgoaceae	*Ginkgo biloba*	129	
Gymnosperms–Pinaceae	*P. taeda*	243	
Angiosperm dicotyledons–Solanaceae	*Solanum lycopersicum*	162	
Angiosperm dicotyledons–Brassicaceae	*A. thaliana*	123	
Angiosperm dicotyledons–Salicaceae	*P. trichocarpa*	281	[46]
Angiosperms monocotyledons–Poaceae	*O. sativa*	184	[11]

**Table 3:** Gene expression profile and functional characterization of *UGT72*s associated with monolignol glycosylation. ns: not studied./: no phenotype observed.

Gene (Species)	Preferential Expression (by RT-qPCR)	Promoter Activity (by GUS Assay)	Mutation/Silencing	Overexpression	References
*UGT72B1* (*A. thaliana*)	Young stem (6-week-old)	Cortex, xylem and pith of the young stem (about 6-week-old); xylem of the old stem (about 6-week-old)	Mutation: 3-fold more coniferin in young stem; increased (1.6-fold more lignin) and ectopic lignification in floral stems; 4-fold higher S/G ratio; 4-fold thicker secondary cell walls; repression of shoot growth	1.7-fold more coniferin in young stem	[39]
*UGT72E1* (*A. thaliana*)	2-day-old seedling; 14-day-old root; 14-day-old aerial part; 4-week-old leaf; 4-week-old senescent leaf	Root of the seedling (10-day-old); base of trichome (10-day-old); base of the silique	/	2-fold more coniferin in light-grown roots; accumulation of coniferin in leaves	[36,37]
*UGT72E2* (*A. thaliana*)	2-day-old seedling; 14-day-old root	Vascular tissue of the leaf (4-week-old), flower (4-week-old) and seedling (10-day-old)	Silencing: 2-fold less coniferin and syringin in light-grown roots	10-fold more coniferin and 2-fold more syringin in light-grown roots; accumulation of coniferin and syringin in leaves; 6-fold less sinapoyl malate in leaves; accumulation of ferulic acid glucoside in leaves; less susceptible to *Verticillum longisporum*	[36]
*UGT72E3* (*A. thaliana*)	Seedling (2-day-old); root (14-day-old); flower (4-week-old); silique	Vascular tissue of the flower (4-week-old) and seedling (10-day-old)	Mutation: 40% more lignin in xylem and interfascicular fibers of the young part of the floral stem; higher capacity of monolignol incorporation in the cell wall	3-fold more coniferin and 2-fold more syringin in light-grown roots; accumulation of coniferin and syringin in leaves; 15-fold less sinapoyl malate in leaves; accumulation of ferulic acid and sinapic acid glucosides in leaves	[36,37,40]
*PtGT1* (*P. tomentosa*)	Upper stem (2-month-old)	ns	ns	Early flowering (40% less leaves at bolting); 60% more lignin in stem (when expressed in *Nicotiana tabacum*)	[38]
*UGT72AZ1* (*P. tremula × P. alba*)	Phloem of the stem (4-month-old)	Phloem in the stem and leaf (4-month-old)	ns	Accumulation of coniferin and syringin in leaves	[61]
*UGT72AZ2* (*P. tremula × P. alba*)	Young root (4-month-old)	Cortex, phloem and differentiating xylem in the root (4-month-old)	ns	Accumulation of coniferin in leaves	[61]
*UGT72B37* (*P. tremula × P. alba*)	Secondary xylem of the stem (4-month-old)	Xylem of the stem (4-month-old)	ns	/	[61]
*UGT72B39* (*P. tremula × P. alba*)	Secondary xylem of the stem (4-month-old); young root (4-month-old)	Xylem of the stem (4-month-old)	ns	/	[61]

**Table 4:** Gene expression profile and functional characterization of *UGT72*s associated with flavonoid homeostasis. ns: not studied./: no phenotype observed.

UGT72 (Species)	Preferential Expression (RT-qPCR)	Promoter Activity (GUS Assay)	Mutation/Silencing	Overexpression	References
UGT72L1 (*M. truncatula*)	ns	Expressed in *A. thaliana*: junction hypocotyl-root; base of the rosette leaves; tip of the cotyledon; leaf trichome; mid-rib of rosette leaves; peduncles of siliques and inflorescence; immature seed	Mutation: 30% less epicatechin, epicatechin 3′-*O*-glucoside; 50% less extractable proanthocyanidins in the seeds	Hairy root: 100% more extractable proanthocyanidins; 25% more non-extractable proanthocyanidins; 40% less anthocyanins	[95]
UGT72A2 (*P. tremula × P. alba*)	Young stem; young leaf	Primary xylem of the stem	Silencing: leaf yellowing and necrosis; in leaves: 50% higher lipid peroxidation; 30% less total flavonoids; 40% less anthocyanins; 20% less phenolics; 5-fold more soluble proanthocyanidins; 3-fold more insoluble proanthocyanidins; 3-fold less soluble peroxidase activity; 2-fold lower NADPH/NADP^+^ ratio; higher tolerance to methyl viologen	30% more total flavonoids in leaf	[27,61]
UGT72AD1 (*L. japonicus*)	Seed (20 days after pollination)	ns	ns	Hairy root: 1.8-fold more flavonol. Expressed in *A. thaliana*: 2-fold more flavonoid and flavonol in seedling; inhibition of root growth	[66]
UGT72Z2 (*L. japonicus*)	Seed (16 days after pollination)	ns	ns	Hairy root: 1.6-fold more flavonol. Expressed in *A. thaliana*: 1.7-fold more flavonoid and 1.5-fold more flavonol in seedling; inhibition of root growth	[66]

## Data Availability

Not applicable.
